# Chimeric antigen receptor T (CAR-T) cells expanded with IL-7/IL-15 mediate superior antitumor effects

**DOI:** 10.1007/s13238-019-0643-y

**Published:** 2019-06-27

**Authors:** Jianxia Zhou, Liyuan Jin, Fuping Wang, Yuan Zhang, Bing Liu, Tongbiao Zhao

**Affiliations:** 1 State Key Laboratory of Reproductive Biology, Institute of Zoology, Chinese Academy of Sciences, Beijing, 100101 China; 2 Graduate University of Chinese Academy of Sciences, Beijing, 100049 China; 3 State Key Laboratory of Proteomics, Translational Medicine Center of Stem Cells, 307-Ivy Translational Medicine Center, Laboratory of Oncology, Affiliated Hospital, Academy of Military Medical Sciences, Beijing, 100071 China; 4 College of Life Sciences, Hebei University, Baoding, 071002 China

**Dear Editor,**

Genetic engineering of T cells to express chimeric antigen receptors (CARs) is an efficient approach for clinical therapy of hematological malignancies (Kuwana et al., 1987; Eshhar et al., 1993; Barrett et al., 2014). The CARs endow T cells with the ability to recognize specific antigens and bind them in an MHC-independent manner, thereby overcoming some of the mechanisms that mediate tumor immune escape. In addition, by providing co-stimulatory signals, CARs endow T cells with enhanced cytotoxicity and persistence compared with primary T cells. A typical CAR comprises a single-chain variable fragment (scFv) derived from a monoclonal antibody (mAb) for antigen recognition and signaling domains for co-activation (Eshhar et al., 1993; Sadelain et al., 2013).

To date, CAR-T cell therapy has been most effective in immunotherapy of CD19^+^ B cell acute lymphoblastic leukemia, with a complete response in more than 75% of cases (Sadelain et al., 2013). However, there are still some challenges for CAR-T-mediated treatments. Side effects like off-targeting, cytokine release syndrome (CRS) and neuronal toxicities have been reported, and these may induce lethal responses (Morgan et al., 2010; Park et al., 2011). In addition, no response, incomplete tumor regression, and tumor recurrence were also observed after CAR-T treatment. For example, 10%–20% of patients were non-responsive to CD19 CAR-T clinical therapy (Lee et al., 2015; Park et al., 2018). Even in cases with a complete response, about 50% of them suffered tumor recurrence in one year, and one third of them had a CD19^+^ relapse (Maude et al., 2018; Orlando et al., 2018). These disappointing results are associated with early CAR-T cell disappearance or poor cell function, which leads to incomplete tumor regression or loss of long-term antitumor effects.

Cytokines are important factors for T cell development and homeostasis. In addition to the TCR and costimulatory receptors, cytokines provide stimulatory signals for full T cell activation, and have pleiotropic effects on T cell proliferation, differentiation and function. Currently, IL-2 is the main cytokine used to culture cells for adoptive cell therapy, as it plays an important role in the proliferation and functional effect of T cells. However, T cells cultured with IL-2 are phenotypically heterogeneous, being predominantly composed of effector memory cells which have sufficient functional effect but are sensitive to death.

IL-7 has a critical role in the development and maturation of T cells. It promotes the generation of naïve and central memory T cell subsets and regulates their homeostasis. IL-15 mediates the formation and homeostasis of CD8 memory T cells. It has been reported that IL-7 and IL-15 are able to instruct T cells toward memory stem-like phenotypes, which are less differentiated and have a superior capacity for expansion and survival (Cieri et al., 2013). Here, we systematically compared the effects of IL-7/IL-15 and IL-2 on the expansion, apoptosis and anti-tumor responses of CAR-T cells.

We first constructed the anti-CD19 CAR (19BB-CAR) using an anti-CD19 mAb (clone FMC63)-derived scFv linked to the *CD8α* hinge and transmembrane regions, followed by a *4-1BB* intracellular signaling domain and the *CD3ζ* signaling moiety. The 19BB-CAR and enhanced green fluorescent protein (eGFP) sequences were ligated and subcloned into the lentiviral vector FUW with a substitutive *EF1α* promoter (Fig. S1A). The cultured primary T cells were stimulated with anti-CD3/anti-CD28 Dynabeads and cytokine IL-2 before transduction with 19BB-CAR lentiviral particles. Using Protein L binding to the variable immunoglobulin light chains of the CAR, we found that CAR expression is directly correlated to eGFP expression (Fig. S1B). The CAR was highly expressed in IL-2-cultured T cells three days after infection (Fig. S1C). The CAR-T cells were expanded 100-fold in 2 weeks under IL-2 stimulation (Fig. S1D).

To test the specificity of 19BB-CAR-T cells, we co-incubated them with two human leukemia cell lines, Raji (CD19^+^) and K562 (CD19^−^). The secretion of IL-2, IFN-γ and TNF-α by 19BB-CAR-T cells was significantly increased upon co-incubation with CD19^+^ Raji but not CD19^−^ K562 cells (Fig. S1E). Accordingly, cytotoxicity assays showed that 19BB-CAR-T cells specifically lysed CD19^+^ Raji but not CD19^−^ K562 cells (Fig. S1F). These data suggest that 19BB-CAR-T cells specifically recognize the CD19 molecule.

To evaluate the anti-tumor effects of 19BB-CAR-T cells cultured using IL-2 *in vivo*, CD19^+^ Raji cells labeled with fluorescent luciferase fusion protein were engrafted to immunodeficient mice for lymphoma formation. Then human T cells transduced with 19BB-CAR or GFP vectors were infused into the mice (Fig. S1G). Mice receiving 19BB-CAR-T cells showed effective tumor regression, while mice infused with T cells harboring empty vector had progressive tumor growth (Fig. S1G–H). Long-term monitoring showed that mice infused with 19BB-CAR-T cells had a significantly higher survival rate and longer survival period compared with mice receiving empty-vector T cells (Fig. S1I). These data provide evidence that 19BB-CAR-T cells can effectively remove tumor cells *in vivo*. However, lymphoma recurrence was observed in some mice treated with 19BB-CAR-T cells, and nearly half of the 19BB-CAR-T mice died within 60 days of infusion due to the tumor burden. These phenotypes are consistent with clinical data, which calls for optimization of CAR-T cells for more efficient tumor killing.

Proliferation and apoptosis are two major aspects to be considered for *in vitro* expansion of CAR-T cells. In the two-week *in vitro* culture assays, we found that the 19BB-CAR-T cells were more efficiently expanded with IL-7/IL-15 than with IL-2 (Fig. 1A). Satisfactorily, there were no differences in the CAR transduction efficiency of T cells cultured in IL-2 or IL-7/IL-15 (Fig. S2). 19BB-CAR-T cells cultured with IL-7/IL-15 showed higher proliferation and a lower apoptosis rate compared to cells cultured with IL-2 (Fig. 1B and 1C). Consistent with this phenotype, the expression of the anti-apoptosis protein BCL-2 is higher in 19BB-CAR-T cells cultured with IL-7/IL-15 than with IL-2 (Fig. 1D). Together, these data suggest that IL-7/IL-15 provide a better environment than IL-2 for CAR-T cell expansion.

**Figure 1:**
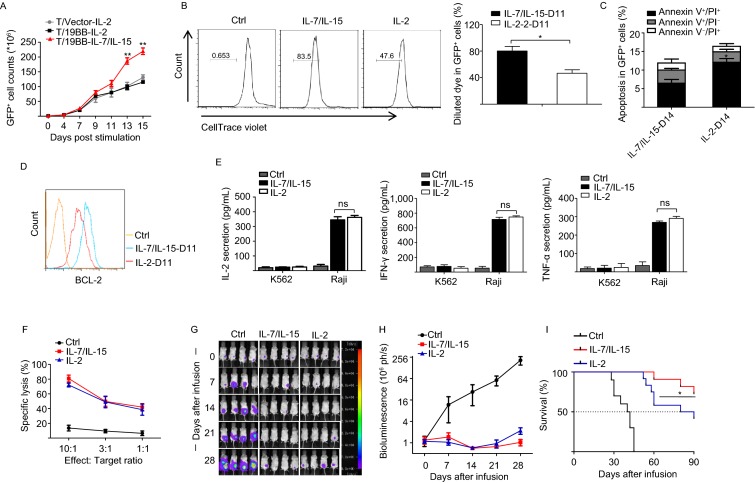
**IL-7/IL-15 supplements induce increased proliferation of 19BB-CAR-T cells and mediate superior anti-tumor effects*in vivo***. (A) *Ex vivo* proliferation of 19BB-CAR-T cells following stimulation with anti-CD3/CD28 antibodies and cytokines IL-7/IL-15 or IL-2. The results are from 4 independent experiments, **P < 0.01. (B) CytoTell Blue staining to detect the proliferation of 19BB-CAR-T cells cultured with IL-7/IL-15 or IL-2 at day 11. The results are from 4 independent experiments, *P < 0.05. (C) 19BB-CAR-T cells cultured with IL-7/IL-15 have a lower apoptosis rate than cells cultured with IL-2. Annexin V and PI were used to determine the proportion of apoptotic cells after a two-week culture. Data are shown as mean ± SEM from 3 independent experiments. *P < 0.05. (D) 19BB-CAR-T cells cultured with IL-7/IL-15 show increased expression of the anti-apoptosis protein BCL-2 compared to cells cultured with IL-2. Cells were cultured with IL-7/IL-15 or IL-2 for 11 days and then analyzed by flow cytometry using anti-BCL-2 antibody. (E) ELISA detection of IL-2, IFN-γ and TNF-α secretion by 19BB-CAR-T cells expanded with IL-7/IL-15 or IL-2. The cells were stimulated by Raji or K562 cells for 24 hours. Data are presented as mean ± SEM from 3 independent experiments. ns, not significant. (F) 19BB-CAR-T cells expanded with IL-7/IL-15 or IL-2 have similar cytotoxicity. Data are presented as mean ± SEM from 3 independent experiments. (G) Representative images of Raji/LUC tumor regression in mice treated for 4 weeks with19BB-CAR-T cells expanded with IL-7/IL-15 or IL-2, *n* = 4 per group. (H) Mean photon flux ± SEM of bioluminescent signals in mice receiving infusions of 19BB-CAR-T cells expanded with IL-7/IL-15 or IL-2. Data are from 3 independent experiments, *n* = 4 per group. (I) Survival curves of mice receiving 19BB-CAR-T cells expanded with IL-7/IL-15 or IL-2. Data are from 3 independent experiments, *n* = 4 per group. *P < 0.05 (IL-7/IL-15 vs. IL-2)

We next investigated the functional properties of CAR-T cells expanded in IL-7/IL-15 or IL-2. The results showed no significant difference in immune cytokine release (IL-2, IFN-γ, TNF-α) or specific lysis (Fig. 1E and 1F). We also investigated the cytokine secretion and cytotoxicity of 19BB-CAR-T cells after serial antigen stimulation to mimic tumor encounter *in vivo*, and found no significant differences between the two culture systems (Fig. S3A–C).

We then infused the IL-7/IL-15- or IL-2-expanded 19BB-CAR-T cells into mice with lymphoma for detection of tumor suppression effects. The antitumor effects were similar in the first 3 weeks. However, the 19BB-CAR-T cells expanded in IL-7/IL-15 showed superior anti-tumor activity, and the long-term survival of the tumor burden mice was significantly improved (Fig. 1G–I).

According to their surface expression of CD45RA and CD62L, primary T cells are divided into four differentiation states: naïve T cells (T_N_) (CD45RA^+^CD62L^+^), central memory T cells (T_CM_) (CD45RA^−^CD62L^+^), effector memory T cells (T_EM_) (CD45RA^−^CD62L^−^), and CD45RA^+^ effector memory T cells (T_RAEM_) (CD45RA^+^CD62L^−^) as reported (Cieri et al., 2013). We found that CD8^+^ CAR-T cell expansion was enhanced during culture with IL-7/IL-15 (Fig. 2A). IL-7/IL-15 induced an increase of the CD8^+^ naïve T cell and central memory T cell populations, while IL-2 enhanced the CD8^+^ effector memory T cell population *in vitro* (Fig. 2B–C). These data indicate that IL-7/IL-15-expanded 19BB-CAR-T cells, which have undergone limited differentiation, may engraft into tumor-bearing mice more efficiently.

**Figure 2:**
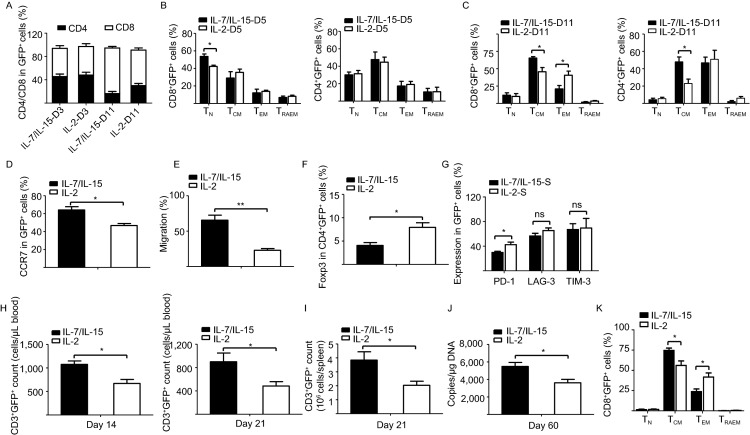
**19BB-CAR-T cells cultured with IL-7/IL-15 show a superior antitumor phenotype*in vitro*and enhanced grafting efficiency after infusion into tumor-bearing mice**. (A) 19BB-CAR-T cells cultured with IL-7/IL-15 generate a higher percentage of CD8^+^ T cells compared to cells cultured with IL-2. Bars show the distribution of CD4^+^ and CD8^+^ T cells in 19BB-CAR-T cells cultured with IL-7/IL-15 or IL-2 at day 3 (D3) and day 11 (D11). Data are presented as mean ± SEM from 4 independent experiments. (B) 19BB-CAR-T cells cultured with IL-7/IL-15 generate a higher percentage of CD8^+^ naïve cells (T_N_) compared to cells cultured with IL-2. Expression of CD45RA and CD62L was assessed by flow cytometry analysis of 19BB-CAR-T cells cultured with IL-7/IL-15 or IL-2 at day 5 (D5). The percentages of T_N_ (CD45RA^+^CD62L^+^), T_CM_ (CD45RA^−^CD62L^+^), T_EM_ (CD45RA^−^CD62L^−^), and T_RAEM_ (CD45RA^+^CD62L^−^) in CD8^+^ lymphocytes (left) and CD4^+^ lymphocytes (right) are shown. Results are presented as mean ± SEM from 4 independent experiments, *P < 0.05. (C) 19BB-CAR-T cells expanded with IL-7/IL-15 generate a larger population of central memory T cells during culture. The percentages of T_N_ (CD45RA^+^CD62L^+^), T_CM_ (CD45RA^−^CD62L^+^), T_EM_ (CD45RA^−^CD62L^−^), and T_RAEM_ (CD45RA^+^CD62L^−^) in CD8^+^ lymphocytes (left) and CD4^+^ lymphocytes (right) in 19BB-CAR-T cells cultured with IL-7/IL-15 or IL-2 at day 11 are shown. Data are presented as mean ± SEM from 4 independent experiments, *P < 0.05. (D) IL-7/IL-15 enhance CCR7 expression compared to IL-2. The expression of CCR7 on 19BB-CAR-T cells cultured with IL-7/IL-15 or IL-2 at day 11 was detected by flow cytometry. Results are presented as mean ± SEM from 4 independent experiments, *P < 0.05. (E) 19BB-CAR-T cells cultured with IL-7/IL-15 show higher migration ability compared to cells cultured with IL-2. Results are presented as mean ± SEM from 3 independent experiments, **P < 0.01. (F) IL-7/IL-15 decreases the Foxp3^+^CD4^+^ T cell population. The expression of Foxp3 in 19BB-CAR-T cells cultured with IL-7/IL-15 or IL-2 at day 11 was detected by flow cytometry. Results are shown as mean ± SEM from 4 independent experiments, *P < 0.05. (G) The percentage of 19BB-CAR-T cells expressing the inhibitory receptor PD-1 is lower after culture with IL-7/IL-15 than with IL-2. The expression of PD-1, LAG-3 and TIM-3 on 19BB-CAR-T cells cultured with IL-7/IL-15 or IL-2 was determined by flow cytometry. Results are presented as mean ± SEM from 3 independent experiments, *P < 0.05. (H) IL-7/IL-15 increase the survival rate of 19BB-CAR-T cells in peripheral blood of tumor-bearing mice. CD3^+^GFP^+^ cells were detected in peripheral blood by flow cytometry in lymphoma-bearing mice at days 14 and 21 after infusion. Results are presented as mean ± SEM from 5 independent experiments, *P < 0.05. (I) IL-7/IL-15 enhance survival of 19BB-CAR-T cells in spleen of tumor-bearing mice after infusion. CD3^+^GFP^+^ cells were detected by flow cytometry in the spleen of lymphoma-bearing mice at day 21 after infusion. Results are shown as mean ± SEM from 5 independent experiments, *P < 0.05. (J) Copy numbers of 19BB-CAR vector per microgram genomic DNA in the peripheral blood of mice receiving 19BB-CAR-T cells at day 60 after infusion. Results are shown as mean ± SEM from 4 independent experiments, *P < 0.05. (K) IL-7/IL-15 maintain the CD8^+^ T_CM_ (CD45RA^-^CD62L^+^) population in 19BB-CAR-T cells from peripheral blood in tumor-bearing mice. Flow cytometry results are presented as mean ± SEM from 4 independent experiments, *P < 0.05

Chemokine receptor CCR7 is involved in lymph-node homing of T_N_ and T_CM_ cells, as well as lymph-node migration of dendritic cells. The expression levels of chemokine receptors CCR7 and CXCR4 are higher in 19BB-CAR-T cells expanded in IL-7/IL-15 than in IL-2 (Figs. 2D and S4). Accordingly, in a gradient of chemokine CCL21, 19BB-CAR-T cells cultured in IL-7/IL-15 showed enhanced migration ability compared to cells cultured in IL-2 (Fig. 2E).

Regulatory T (Treg) cells play an important role in immunosuppression. We found that IL-2 mediated a smaller increase of the CD4^+^Foxp3^+^ 19BB-CAR-T cell population than IL-7/IL-15 during *in vitro* expansion of T cells (Fig. 2F). In addition, we found decreased expression of PD-1 in 19BB-CAR-T cells cultured in IL-7/IL-15 compared to IL-2 upon serial antigen stimulation by Raji cells, which indicates that IL-7/IL-15 decrease CAR-T cell exhaustion (Fig. 2G). These data provide supporting evidence that IL-7/IL-15 induce a superior anti-tumor activity in 19BB-CAR-T cells compared to IL-2.

We further evaluated the survival and memory T cell phenotype of 19BB-CAR-T cells cultured with different cytokines after infusion into tumor-bearing mice. 19BB-CAR-T cells cultured with IL-7/IL-15 showed enhanced engraftment in mice compared to cells cultured with IL-2 (Fig. 2H–J). After infusion into mice, 19BB-CAR-T cells cultured in IL-7/IL-15 generated more CD8^+^ central memory T cells than CAR-T cells treated with IL-2, while cells cultured in IL-2 generated more CD8^+^ effector memory T cells than cells cultured in IL-7/IL-15 (Fig. 2K). This is consistent with the *in vitro* results. These data provide evidence that CAR-T cells cultured in IL-7/IL-15 have superior anti-tumor activity *in vivo*.

Generating optimized CAR-T cells *in vitro* is an important strategy to enhance the clinical efficacy of CAR-T cells in cancer immunotherapy. Recently, Xu et al. reported that IL-7/IL-15 are better than IL-2 for preserving the CD8^+^CD45RA^+^CCR7^+^ population in *ex vivo*-cultured CAR-T cells, and endow the CAR-T cells with superior proliferation and survival capability upon serial antigen stimulation (Xu et al., 2014). Another study showed that IL-7/IL-15 instruct the expansion of CD62L^+^CD45RA^+^ memory T cells from naïve precursors (Cieri et al., 2013). In contrast, we found that IL-7/IL-15 promotes CAR-T cell proliferation directly without antigen stimulation *in vitro*, and the level of apoptosis is low. CAR-T cells cultured with IL-7/IL-15 expanded around 2-fold more within two weeks than cells cultured with IL-2, which will favor the generation of CAR-T cells for certain patients whose lymphocytes have limited expansion ability.

In conclusion, we systematically compared the effects of IL-7/IL-15 and IL-2 on CAR-T cell culture, and demonstrated that CAR-T cells expanded in the presence of IL-7/IL-15 showed enhanced proliferation and superior antitumor activity. IL-7/IL-15 selectively expanded naïve and central memory T cells, which help CAR-T cell engraftment in tumor-bearing mice. Apart from IL-2, IL-7 and IL-15, many other cytokines are important for T cell development, differentiation and function. IL-12 is involved in the differentiation of naïve Th0 cells into Th1 cells, and augments the activity of cytotoxic T cells. IL-18 regulates the immune response by enhancing the secretion of IFN-γ and augmenting cytolytic activity. These cytokines could be potentially investigated for optimization of CAR-T expansion.

## Electronic supplementary material

Below is the link to the electronic supplementary material.

Supplementary material 1 (PDF 396 kb)
