# Supplementary material for: Chimeric antigen receptor T (CAR-T) cells expanded with IL-7/IL-15 mediate superior antitumor effects
Source: Protein Cell. 2019 Jun 27;10(10):764–9. doi: 10.1007/s13238-019-0643-y (PMC6776495; doi:10.1007/s13238-019-0643-y)

Supplementary material

Materials and Methods

CAR preparation

To target CD19, we designed a second-generation CAR construct, referred to as pFUW-EF1α-19BB-CAR, in the lentiviral FUW vector. The construct includes scFv derived from the mouse monoclonal antibody FMC63 linked to the CD8 hinge and transmembrane domain, the cytoplasmic portions of 4-1BB. and the signaling moiety of the CD3ζ chain. The EF1α promoter was used for strong CAR expression. The CAR sequences were synthesized at Sangon Technology (Shanghai, China) and cloned into the lentiviral vector. The CAR sequences were followed with a fluorescent marker P2A-eGFP to detect transfection efficacy. The FUW vector harboring P2A-eGFP sequences was used as a negative control. Lentiviral particles were generated in 293T-packaging cells by calcium phosphate transfection of pMD2.G plasmid, psPAX2 plasmid and the vector plasmid. The transfected cells were incubated at 37 °C for 12 h, and then in fresh medium for another 48 h. Supernatants containing lentiviral particles were collected and filtered through a 45 μm filter, and then concentrated by ultracentrifugation at 20000 rpm for 120 min at 4°C. The final viral pellets were resuspended in X-VIVO 15 medium (Lonza) and stored at -80 °C.

CAR-T cell generation and cell cultures

We isolated peripheral blood mononuclear cells (PBMCs) by Ficoll-Paque PLUS gradient centrifugation (GE Healthcare) from peripheral blood of healthy volunteer donors. Primary T cells were sorted with a Pan T Cell Isolation Kit (Miltenyi Biotech) through negative

selection from PBMCs according to the manufacturer’s instructions. T cells were cultured in X-VIVO 15 medium supplemented with 5% heat-inactivated fetal bovine serum (Hyclone), 2 mM glutamine (Gibco), and 1 mM sodium pyruvate (Gibco) at a concentration of 10^6 cells/mL. T cells were stimulated with magnetic beads coated with agonist antibodies against CD3 and CD28 (Invitrogen Life Technologies) at a bead-to-T cell ratio of 1:1 and recombinant human IL-2 or IL-7/IL-15 (PeproTech) for 36 hours. T cells were then transduced with 19BB-CAR by adding lentiviral particles and polybrene (8 μg/mL) (Sigma) for 24 hours. For expansion, the 19BB-CAR-T cells were cultured in X-VIVO 15 medium with (1) IL-2 (200 U/mL), or (2) IL-7 (10 ng/mL) plus IL-15 (10 ng/mL) in 6-well non-tissue culture plates (Corning) for about two weeks. The 19BB-CAR-T cells were incubated at 37 °C with 5% CO_2, and the culture medium and cytokines were replaced every two days. For serial antigen exposure, CAR-T cells were cocultured with Raji cells *in vitro* at an effector-to-target ratio of 1:1 for two days. Three sequential rounds of coculture were carried out.

The human tumor cell lines Raji and K562 were cultured in RPMI 1640 (Gibco) supplemented with 10% heat-inactivated fetal bovine serum (Hyclone), 2 mM glutamine (Gibco), 0.1 mM nonessential amino acids (Gibco), and 1% Penicillin/Streptomycin (Gibco). The packaging cell line 293T was maintained in DMEM (Gibco) supplemented with 10% heat-inactivated fetal bovine serum, 2 mM glutamine, 0.1 mM nonessential amino acids, 1 mM sodium pyruvate, and 1% Penicillin/Streptomycin.

Flow cytometry and antibodies

Cell surface marker expression was determined using a flow cytometer. CAR-T cells were

stained with fluorescently-labeled antibodies against cell surface markers as follows: CD3-Percp/Cy5.5, CD4-APC, CD8-Brilliant Violet 510 (all from Biolegend), CD45RA-PE, CD62L-PE/Cy7, CCR7-APC, CXCR4-APC, IL7R-Brilliant Violet 421, CD95-efluor 450, CD25-efluor 450, CD27-APC, CD28-PE (all from eBioscience). Cells were washed with PBS and counted by Trypan Blue staining. A total of 10^6 cells were suspended in PBS with 1% BSA for 15 minutes, and then incubated with the specific antibody for 30 minutes at 4°C. For intracellular staining, CAR-T cells were fixed and permeabilized by Flow Intracellular Staining Reagents (eBioscience), and then stained with BCL-2 antibody (Bioss). For intranuclear staining, CAR-T cells were fixed and permeabilized using Foxp3/Transcription Factor Staining Buffer Set (eBioscience) and then stained with Foxp3 antibody (Abcam) in perm buffer. Samples were washed twice with PBS and suspended in flow cytometry staining buffer (eBioscience). For analysis of cell division and apoptosis in CAR-T cells, the fluorescent dye CytoTell blue (AAT Bioquest) and Alexa Fluor 647-Annexin V/PI reagent (YEASEN) were used respectively to stain T cells according to the manufacturer’s instructions. Data were acquired using a FACS (BD FACS AriaIII) and analyzed by FlowJo 7.6.1 software.

To measure CAR-T cell phenotypes in mouse peripheral blood, a total of 500 μL venous blood was collected into K2EDTA anticoagulation tubes from the orbital venous plexus and PBMCs were separated with Ficoll-Hypaque gradient centrifugation. To measure the absolute CAR-T cell numbers in mouse blood, 50 μL venous blood was collected from the orbital venous plexus. BD Trucount tubes (BD Bioscience) and antibodies were used to calculate the absolute numbers and percentage of CAR-T subsets according to the

manufacturer's instructions. To evaluate the numbers of CAR-T cells in mouse spleen, the spleen tissues were isolated and ground into single cells. After treatment with erythrocyte lysate, the cells were diluted in medium and the absolute number of cells was determined with BD Trucount tubes and flow cytometry.

Cytokine secretion

Before the experiment, the anti-CD3/CD28 microbeads were removed magnetically and the plain X-VIVO 15 medium wasused for CAR-T cell stimulation. CAR-T cells were cultured with target tumor cells at an effector-to-target ratio of 1:1 ($5*10^4$ cells each) in 96 well round bottom plates (Nunc) for 24 hours. The cells were mixed completely, and the treatments were performed in triplicate. Then supernatants were collected to assess the secretion of cytokines (IL-2, IFN-γ, TNF-α) by enzyme-linked immunosorbent assay (ELISA) (eBioscience) according to the manufacturer's instructions. The cytokine concentration was quantified from an eight-point standard curve. Data were acquired on a Multiscan Spectrum (Luminex) and concentrations were obtained in units of pg/mL.

Cytotoxicity assay

Before the experiment, the anti-CD3/CD28 microbeads were removed magnetically. We determined the specific cytotoxicity of CAR-T cells toward target cells by lactate dehydrogenase (LDH) assay using the CytoTox 96 nonradioactive cytotoxicity kit (Promega) at effector-to-target ratios of 10:1, 3:1, and 1:1 for 4 hours. The cells were mixed in 96 well round bottom plates and analyzed in triplicate. The target cells were suspended in medium with a concentration of $1*10^4$/50 μL and the effector cell numbers were set according to the ratio. Supernatants were collected for detection of LDH release

according to the manufacturer's instructions.

***In vitro* migration assay**

We added no-serum medium containing CCL21 (400 ng/mL) in the lower chamber of trans-well plates (Corning). CAR-T cells were added into the upper chamber in no-serum medium for six hours. The number of CAR-T cells that migrated toward the chemokine CCL21 was analyzed by cell counting with Trypan Blue staining.

***In vivo* antitumor study**

All animal studies were carried out under protocols approved by the Institutional Animal Care and Use Committee. 8-week-old male NOD-Prkdc^{scid} IL-2rg^{null} (NPG) mice (VITALSTAR, Beijing, China) were used for human lymphoma xenografts. 5*10^5 CD19$^+$ Raji cells were transduced with a construct expressing a green fluorescent protein-firefly luciferase fusion protein (GFP/LUC) for bioluminescence signaling, then injected into mice via intraperitoneal injection in a volume of 200 µL PBS. Five days later, mice were injected D-luciferin (YEASEN) at a dose of 150 mg/kg, and imaged after 15 minutes by a Luminescence imager (Berthold Technologies). The lymphoma size was measured according to the bioluminescence signal in units of photons/s. Mice with equal tumor burden (1~5×10^6 photons/sec) were selected and randomized to different treatment groups. 1×10^7 CAR-T cells expanded in IL-7/IL-15 or IL-2 were administered to mice by intravenous infusion. Four days later, the same dose of CAR-T cells was infused again. Tumor burden was monitored once per week by *in vivo* bioluminescence imaging. Survival curves were recorded and analyzed through GraphPad Prism Software.

Quantitative real-time PCR

Peripheral blood was collected from the orbital venous plexus and genomic DNA was extracted with a DNA Blood Midi Kit (TIANGEN). To detect the integrated CAR sequence, qPCR analysis was performed with specific primers on 100 ng genomic DNA as template with SYBR Green dye. A standard curve with ten points was generated to determine the copy number/µg DNA. The calculation was performed as described previously. Primers for quantitative real-time PCR were designed as followed: GAPDH-Forward: 5'-GTCAAGGCTGAGAACGGGAA-3'; GAPDH-Reverse: 5'-AAATGAGCCCCAGCCTTCTC-3'; CD19-CAR-Forward: 5'-ACATCCTCCCTGTCTGC-3'; CD19-CAR-Reverse: 5'-CCACTGCCACTGAACC-3'.

Statistical analysis

The mean ± S.E.M. was determined in the experiments. The two-tailed Student t-test was used to determine significant differences between experimental and control groups. Statistical significance was defined as * ($0.01<P<0.05$), ** ($P<0.01$). All data analyses were done using Prism software (GraphPad). Comparison of survival curves was done using the log-rank test. Error bars in graphs represent the SEM.

Supplementary Figure Legends

Supplementary Figure 1. Establishment of 19BB-CAR-T cells.

A, Schematic of the FUW-EF1A-19BB-GFP CAR vector. **B**, The transfection efficiency of the CD19 CAR lentiviral vector (T/19BB) was demonstrated by analyzing the expression of eGFP and protein L. T/Vector is the control (GFP vector). **C**, Primary T cells cultured with CD3/CD28 and IL-2 are efficiently transduced by the 19BB-chimeric antigen receptor

lentivirus construct. **D**, The expansion of CAR-T cells cultured with anti-CD3/CD28 microbeads and IL-2 *in vitro* over time. The results are representative of 4 independent experiments. **E**, 19BB-CAR-T cells are specifically activated by co-culture with $CD19^+$ Raji cells. Secretion of IL-2, IFN-γ and TNF-α was detected by ELISA 24 h after co-culturing. Data are presented as mean ± SEM from 3 independent experiments. **P<0.01. **F**, 19BB-CAR-T cells specifically lyse Raji cells, while having no specific cytotoxic effects on $CD19^-$ K562 cells. Data are presented as mean ± SEM from 3 independent experiments. **P<0.01. **G**, Top: schematic of the CAR-T cell infusion and tumor detection protocol in lymphoma model mice. Bottom: representative images showing 19BB-CAR-T cell-mediated Raji/LUC tumor regression in mice. **H**, Mean photon flux ± SEM of bioluminescent signals in mice receiving 19BB-CAR-T and control cells. **I**, Survival curves of mice receiving 19BB-CAR-T cell and control treatments.

Supplementary Figure 2. No differences in the transduction efficiency of CAR-T cells cultured with IL-7/IL-15 or IL-2.

The graph shows the transduction efficiency of $CD4^+$ and $CD8^+$ subsets of CAR-T cells which were cultured with IL-7/IL-15 or IL-2. Data are presented as mean ± SEM from 3 independent experiments. NS, not significant.

Supplementary Figure 3. No differences in cytokine secretion and cytotoxicity of CAR-T cells cultured with IL-7/IL-15 or IL-2 upon serial antigen stimulation.

A, Schematic of the protocol for serial antigen stimulation of CAR-T cells *in vitro*. **B**, The secretion of cytokines IL-2, IFN-γ and TNF-α by CAR-T cells that were expanded in IL-7/IL-15 or IL-2 then subjected to serial antigen stimulation. Data are presented as mean

± SEM from 3 independent experiments. **C**, Specific cytotoxicity of CAR-T cells expanded in IL-7/IL-15 or IL-2 then serially stimulated with Raji cells at effector-to-target ratios of 1:1, 3:1, and 10:1. Data are presented as mean ± SEM from 3 independent experiments.

Supplementary Figure 4. IL-7/IL-15 induce higher expression of CXCR4 on 19BB-CAR-T cells than IL-2.

Expression of CXCR4 was measured on 19BB-CAR-T cells cultured with IL-7/IL-15 or IL-2 for 11 days. Results are presented as means ± SEM from 4 independent experiments, *$P<0.05$.

Supplementary Figure 1

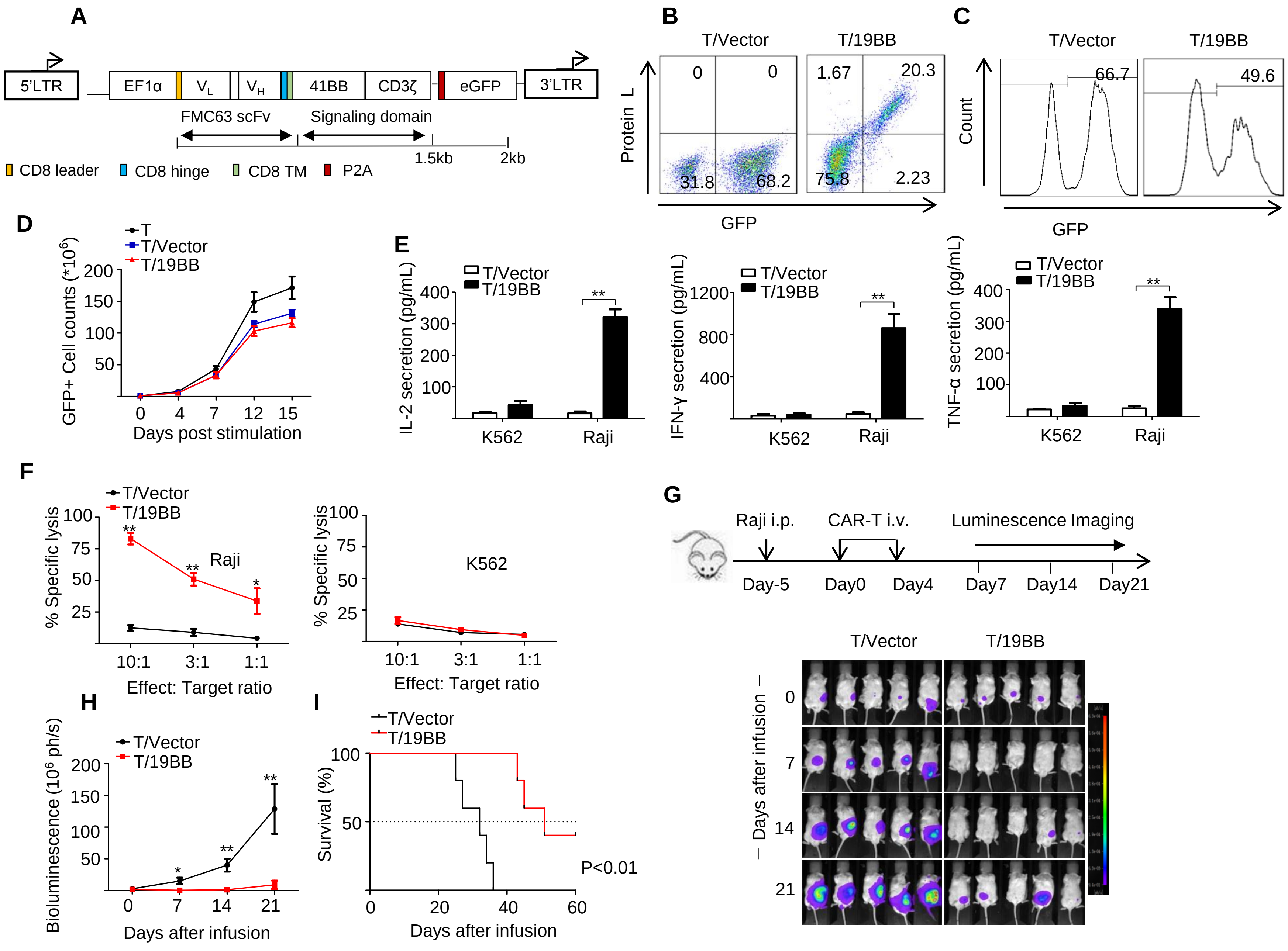

Supplementary Figure 2

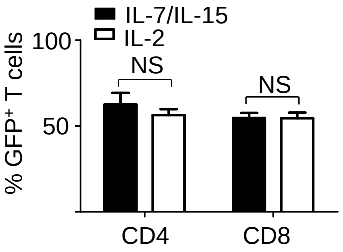

Supplementary Figure 3

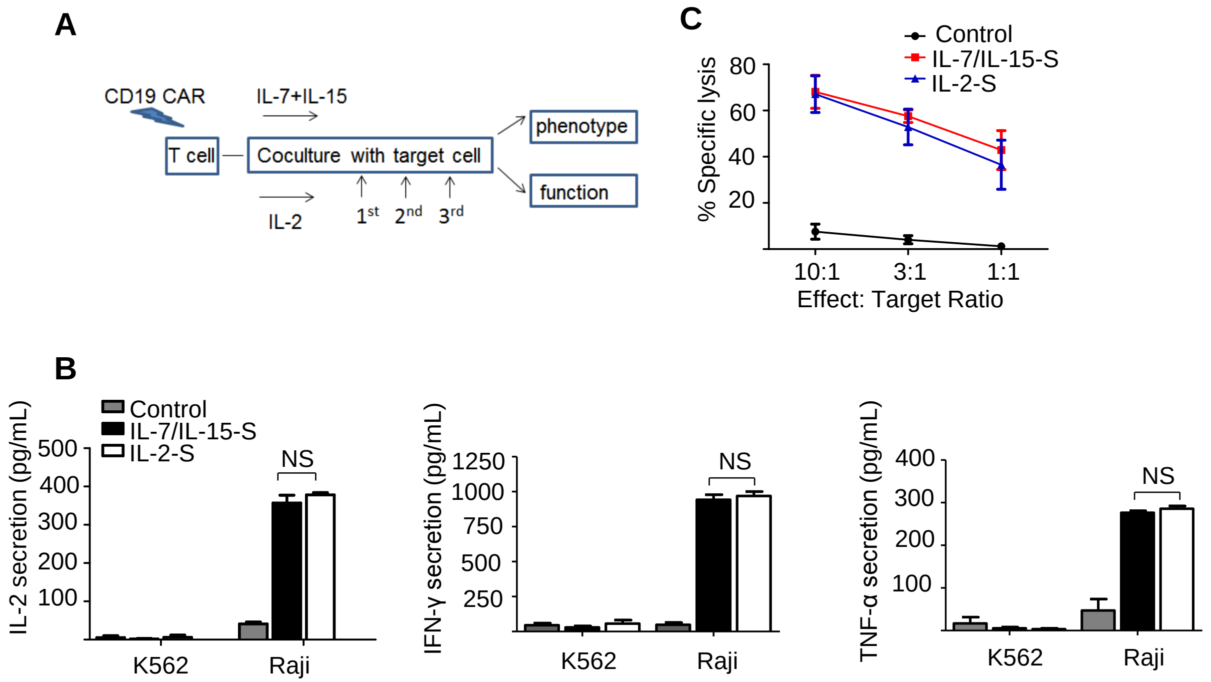

Supplementary Figure 4

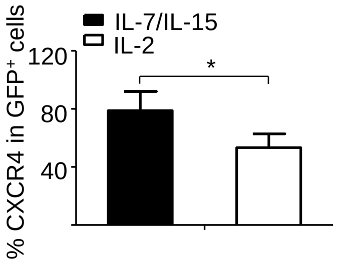

Supplement: Supplementary file 1 — Supplementary material 1 (PDF 396 kb) [file 13238_2019_643_MOESM1_ESM.pdf]
